# Conservation

**Published:** 1918-11

**Authors:** 


### Conservation.

Early in our involvement in the war, the opinion was expressed that, if it were not too long continued, not only the enormous expense but the loss of life might be compensated for. Against the field and camp mortality must be balanced the fact that in the year and a half since war began for us, the approximately three million men in our army would have had somewhere from 25,000 to 35,000 deaths anyhow and an amount of traumatic disability and disease probably not far from the total noted in military statistics. For the future, the protection against typhoid, paratyphoid and small pox by artificial methods, against various infectious diseases which they might or might not have had in civil life but which they have passed through under conditions averaging as favorable at least as in civil life; the sanitary, hygienic, dietetic and physical training; the radical cure of hernia, appendicitis and many other conditions, the sorting out and warning as to future care on the part of defectives of all kinds, will react to produce a lower death rate for a generation. More than this, each soldier not grossly stupid or careless will be a focus of sanitary education and the saving of life and health will undoubtedly be permanent.

While statistics prove that the purchasing power of the ante-bellum dollar is only 59 cents general experience is that by using material on hand, selecting cheaper but not necessarily inferior foods and wares of all kinds, by little thrifts and avoidance of waste, standards of living have been reduced only slightly while wages have risen disproportionately. General prosperity always depends largely on the rapidity with which money circulates. The fact that there is only about $56 of money per capita and that about $200 per capita is being paid to the government, not only indicates that our circulation is good but affords a strong hint as to how it may be maintained at a high pressure. While, especially with regard to railroad fares and to a less degree with regard to other utilities, immediate results have been disappointing, there are reasons for these disappointments and the general expectation is that government ownership of proper utilities will reduce prices or, at least prevent unwarrantable increase. The war has greatly hastened the tendency in this direction and changes should be made with due caution.

The large aggregate value of trifling matters formerly almost entirely wasted, such as tinfoil and paper may properly be devoted to various philanthropies after the war, as the saving is usually too slight to pay as a matter of individual profit and the aggregate can be maintained only by philanthropic duties. More important, the war has shown what many hard-hearted persons, including the present writer have long suspected, that many forms of philanthropy were wasteful and unnecessary and the improvement in economic conditions, elimination of a large amount of drunkenness and loafing, have greatly reduced the burden of philanthropy. It is not impossible that all future philanthropy may be financed on the saving of waste and the voluntary donations and legacies of the very rich.

The war has taught many of us that our scale of living was unnecessarily luxurious. For example any cheap lodging house and beanery are better in comfort, though not in hygienic factors, than the average of camp life on which men grow strong and healthy. It is not desirable that the extreme thrift necessary during war should be continued in peace but the lesson is valuable. The same is true as to the utilization of labor of females, minors and the aged.

Conservative conservation should be the slogan after the war.

